# When is the optimum time for the initiation of early rehabilitative exercise on the postoperative functional recovery of peri-ankle fractures? A network meta-analysis

**DOI:** 10.3389/fsurg.2022.911471

**Published:** 2022-08-16

**Authors:** Ke Zhao, Shilei Dong, Wei Wang

**Affiliations:** ^1^ College of Acupuncture—Moxibustion and Orthopedics, Hubei University of Traditional Chinese Medicine, Wuhan, China; ^2^ Department of Orthopedics, Hubei Provincial Hospital of Traditional Chinese Medicine, Wuhan, China; ^3^ Department of Orthopedics, Affiliated Hospital of Hubei University of Traditional Chinese Medicine, Wuhan, China; ^4^ Institute of Orthopedics, Hubei Province Academy of Traditional Chinese Medicine, Wuhan, China

**Keywords:** peri-ankle fractures, rehabilitative exercise, meta-analysis, postoperative functional recovery, periarticular fractures

## Abstract

**Objective:**

The purpose of this study was to explore the safe and most effective initiation time for the functional recovery of patients with peri-ankle fractures after surgery.

**Method:**

We searched electronic databases, including the Cochrane Library, Embase, PubMed and the reference lists of relevant articles published from inception to October 30, 2021. Two researchers independently performed literature screening and data extraction and evaluated the quality of the included literature using the Newcastle–Ottawa Scale. Network meta-analysis, including consistency testing, publication bias, and graphical plotting, was performed using Stata (v16.0).

**Results:**

A total of 25 articles involving 1756 patients were included in this study. The results of the meta-analysis showed that functional exercise within 2 days after surgery may result in lower VAS scores compared to other techniques (*P* < 0.05). Functional exercise within 12 months may lead to higher AOFAS scores than that of other techniques (*P* < 0.05). The total postoperative complication rate, including deep vein thrombosis, showed no statistically significant differences between any two interventions (*P* > 0.05). The results of the surface under the cumulative ranking (SUCRA) showed that functional exercise within two days postoperatively may have the lowest VAS scores (SUCRA = 82.8%), functional exercise within 1 week postoperatively may have the lowest deep vein thrombosis rate (SUCRA = 66.8%), functional exercise within 10 days postoperatively may have the fewest total postoperative complication rate (SUCRA = 73.3%) and functional exercise within 12 months postoperatively may contribute to the highest AOFAS scores (SUCRA = 85.5%).

**Conclusion:**

The results of this study suggest that initiation of rehabilitation within two days after surgery may be the best time to reduce postoperative pain; rehabilitation interventions within 10 days after surgery may be the optimal time for reducing the total postoperative complication rate, including deep vein thrombosis; and continued functional exercise within 12 months after surgery may steadily and ideally improve the function of the ankle joint.

Systematic Review Registration: doi: 10.37766/inplasy2021.12.0030, identifier: INPLASY2021120030

## Introduction

Peri-ankle fractures are the third most serious musculoskeletal fracture, with a mortality rate as high as 12% during the first year (1). In 2011, 174 cases per 100,000 persons were reported in Germany, accounting for approximately 60% of women aged over 50 years (2). It is predicted that caries of peri-ankle fractures will increase 3-fold by 2030 with the aging population and increasing participation in physical activities (3). The main cause of peri-ankle fracture is sprains and falls, followed by sports injuries. Furthermore, osteoporosis is an also an important cause in elderly individuals (4). The presence of osteoporosis, multiple serious comorbidities and functional independence make treatment a challenge.

According to AO classification, displaced or/and dislocated unstable peri-ankle fractures are recommended to be treated with open reduction and internal fixation (ORIF) to achieve anatomical reconstruction (5). Regardless of whether the patient has conservative or operative treatment, a period of immobilization is essential (6). Ankle joint immobilization is carried out to protect surgical wounds, reduce pain after anesthesia, and minimize mechanical stress (7). The potential contribution of immobilization includes decreased muscle volume and loss of strength, reduced range of mobility, and the consequential influence of function (8). Many patients had good surgical reduction but failed to recover with normal ankle joint function due to the lack of effective postoperative functional exercise.

With the development of multidisciplinary team (MDT)-driven peri-operative care and rehabilitation, many doctors advocate early ankle exercises and weightbearing after surgery to accelerate functional rehabilitation (9). However, the initiation time of postoperative functional exercises and ankle range of motion remain unclear and controversial, which hinders their widespread implementation in the clinic.

An earlier systematic review compared the results of early ankle range of motion and weight-bearing with delayed exercises and concluded that early weightbearing postoperative regimens can improve the stiffness and range of movement of the ankle joint (10). However, there are no systematic reviews on the effects of different postoperative rehabilitation times or direct comparisons of different starting times. Network meta-analysis (NMA) allows for the indirect comparison of different interventions and the selection of the best timing (11). Herein, the purpose of this study was to explore the safe and most effective initiation time for functional recovery of patients with peri-ankle fractures using the NMA method. The study provides valuable information for future treatment and rehabilitation intervention of peri-ankle fractures.

## Methods

This NMA strictly complied with the preferred reporting items for systematic review and meta-analysis (PRISMA) guidelines and Meta-Analyses (PRISMA) guidelines for NMA (12, 13). We have registered this systematic review with INPLASY (CRD2021120030) prospectively and report the results of an included prespecified outcome analysis.

### Inclusion and exclusion criteria

The inclusion criteria were as follows: (1) randomized control trials (RCTs); (2) studies about peri-ankle fractures (fractures or dislocations which affected the ankle mortise and ankle joint stability); (3) the interventions were different functional exercise initiation times for the patients after surgery; and (4) extractable data reporting the total postoperative complication rate, incidence of deep vein thrombosis (DVT), AOFAS scores, and VAS scores of patients.

The exclusion criteria were as follows: (1) duplicate publications, original texts not found; (2) review categories, empirical summaries, case reports, conferences, and meta-analyses; (3) interventions not including the functional exercise initiation time and (4) contraindication for early weightbearing (eg, unstable osteosynthesis, open fracture, pilon fracture other significant comorbidities preventing early mobilization).

### Data sources and search strategies

The PubMed, Cochrane Library, EMBASE and the reference lists of relevant published articles were systematically searched from inception to October 2021. The search strategies were “ankle” OR “distal tibia” OR “distal fibula” OR “talus” OR “medial malleolus” OR “lateral malleolus” OR “posterior malleolus” OR “unicondylar” OR “bimalleolar” OR “trimalleolar” AND “fracture” AND “rehabilitation” OR “training” OR “exercise” OR “weight bearing” AND “randomized” OR “RCT”.

### Study selection

The retrieved literature was imported into the literature management software. To screen the studies, two researchers independently performed the extraction of relevant information, and then the full text of the included studies was analyzed quantitatively according to the inclusion and exclusion criteria. Disagreements between the two researchers were resolved through discussion and negotiation by a third researcher. In addition, the investigators extracted data according to predesigned tables, including the study characteristics (author, year, country), patient characteristics (sample size, male/female, rehabilitation modality, type of fracture, time from injury to operation, intervention initiation time) and outcomes (AOFAS score, VAS score, total postoperative complication rate, DVT incidence, follow-up time).

### Quality assessment

The Cochrane Risk of Bias Assessment Tool was used to assess the quality of the included randomized controlled trials (14), which evaluates the risk of bias in six main areas (random sequence generation, allocation concealment, participant and outcome blinding, incomplete follow-up, selective reporting, and other biases), and the risk of bias distribution was plotted using Revman version 5.3 software.

### Statistical analysis

NMA was performed using Stata (v16.0) software. Comparisons between the different starting times were represented by network plots, with lines between the points indicating direct comparisons and the thickness of the lines indicating the amount of comparison between the two intervention times (15). Local inconsistency tests for direct and indirect comparisons were performed using nodal splits, and *P* < 0.05 was considered to be a local inconsistency. Relative risk (RR) was used for count data, and weighted mean difference (WMD) or standardized standard deviation (SMD) and its 95% confidence interval (CI) were used for measurement data. Intervention efficacy was estimated as the probability of ranking by surface under the cumulative ranking (SUCRA). Publication bias and small sample effects among the included studies were assessed by funnel plots (16). The risk of bias of the included studies was analyzed using Revman, where green, yellow and red in the images represent low risk of bias, unclear and high risk of bias, respectively.

## Results

### Characteristics of the included studies

**Figure 1:**
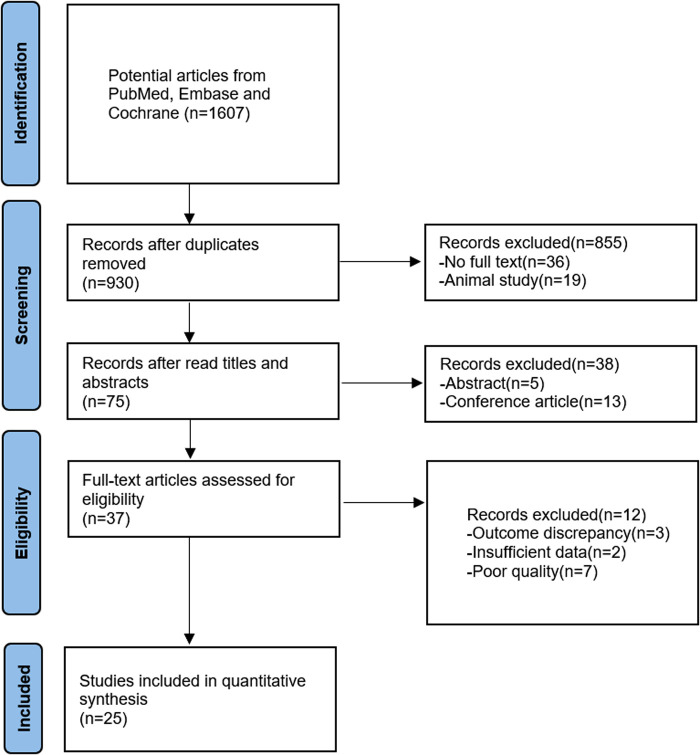
Flow chart of the literature screening process RCT, randomized controlled trial; E, experimental group; C, control group; NA, Not available; lauge-hansen Se, lauge-hansen supination external rotation type; lauge-hansen Pe, lauge-hansen pronation- eversion; RT, rehabilitation training; WB, weight bearing AOFAS scores②VAS scores③total postoperative complication rates④DVT rates.

The detailed article searching and study selection process is listed in Figure 1. After systematic search, a total of 1,607 articles were obtained, and 930 articles were obtained after deweighting by Endnote X9 software. Seventy-five articles were obtained after reading the titles and abstracts and excluding irrelevant studies, noncontrolled experimental studies, conferences, abstracts, meta-analyses, etc. For the remaining articles, three articles with noncompliant outcome indicators, two articles with incomplete data reporting and seven articles were excluded due being considered low-quality articles after reading the full text, and 25 articles with a total of 1,756 patients were finally included. Table 1 lists the basic information of all the included studies.

**Table 1:** Basic characteristics and quality evaluation of the included studies.

Study	Year	Country	Type of study	Sample size (E/C)	Type of fracture	Injury-operation	Initiation	Movement style	Outcomes	Follow-up
Egol K	2000	United Kingdom	RCT	27/28	Weber BC	NA	2w/6w	RT	①	12w
Franke J	2008	Germany	RCT	14/13	Weber B	5d/7d	2d/6w	WB	②	10w
Dogra	1999	United Kingdom	RCT	26/26	Weber B	NA	1d/2w	WB	②③	12w
Anne M	2015	Australia	RCT	106/108	Weber ABC	NA	1w/4w	RT	①③	NA
Christian JP	2005	Germany	RCT	23/23	Weber BC	NA	1d/6w	WB	③	12m
Diederik P	2018	Canada	RCT	36/37	Lauge-Hansen Se2,3,4	NA	10d/6w	WB	③	1y
DiStasio	1994	United Kingdom	RCT	25/31	Weber BC	NA	1w/6w	RT	①③④	6m
Lee DH	2012	Korea	RCT	40/41	Weber BC	NA	2w/6w	WB	①②	24–76m
Hendrik Jansen	2018	Germany	RCT	22/22	Weber BC	7.4 h/8.9h	1d/2d	RT	①②④	12w
Kimmel LA	2012	Australia	RCT	51/53	Weber B	54 h/50.9h	1d/2d	RT	③	NA
Lehtonen	2003	United States	RCT	50/50	Weber AB	30 h/25h	1d /2w	WB	①②③④	6w
Marius M	2020	Norway	RCT	56/57	Weber B	NA	1d/1d	RT	②③④	52w
Michael P	2012	United States	RCT	19/107	Lauge-Hansen Se2,4	NA	1d/8d	WB	③	6w
Michael Z	2021	Germany	RCT	25/20	Weber ABC	8 h/7h	2d/6w	WB	①③④	12m
Mihai V	2007	United Kingdom	RCT	33/29	Lauge-Hansen	NA	1d/6w	RT	①③④	12w
Min W	2019	China	RCT	21/21	Weber ABC	NA	1d/5w	WB	①②	24m
Niloofar D	2016	Canada	RCT	56/54	Weber BC	7 h/6.2h	2w/6w	WB	③	6W
Paolo C	2019	United Kingdom	RCT	32/19	Weber BC	NA	1d/6w	WB	③	12w
Pasquale F	2009	Italy	RCT	22/22	Weber ABC	NA	1d/2w	RT	①③④	10–14y
Torbjorn A	1987	Sweden	RCT	25/28	Lauge-Hansen Se4;Pe34	NA	1d /4w	WB	③	6m
Stöckle U	2000	Germany	RCT	20/20	Weber ABC	NA	2w/6w	RT	①③	6w
William	1989	United States	RCT	32/19	Weber ABC	NA	1d/6w	RT	①③	10–14w
Li	2013	China	RCT	22/21	Weber ABC	NA	1d/6w	RT	①②③	12m
Liao	2010	China	RCT	22/22	Weber BC	NA	1d/3w	RT	①②③④	4.2y
Shi	2020	China	RCT	40/49	Weber ABC	NA	1d/5w	WB	①②	24m

### Quality assessment

**Figure 2:**
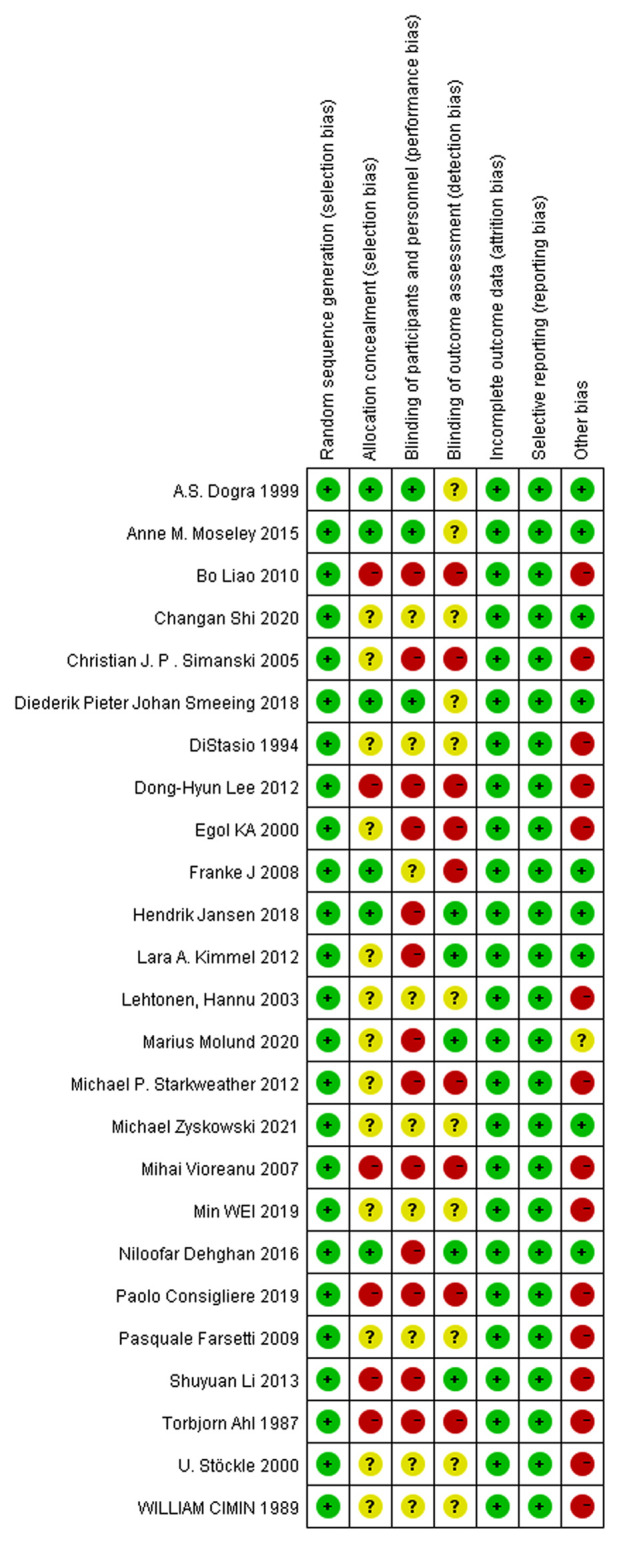
Green: Low risk of bias; yellow: unclear risk of bias; Red: high risk of bias.

In Figure 2, the quality of the 25 articles was good overall, with red indicating high risk, yellow indicating unclear risk, and green indicating low risk in the bias distribution graph. Twenty-five studies were adequately randomized, 6 reported allocation concealment, and five reported blinding of outcome assessment. Thirteen studies did not report blinding of the participants and personnel. All other risks of bias were low.

### Evidence network

**Figure 3:**
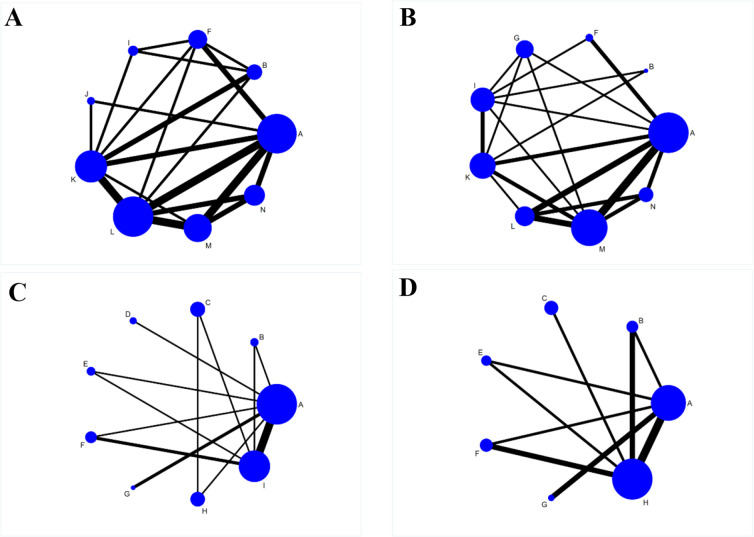
Reticulated evidence diagram of different rehabilitation intervention times after the operation. A: 1 day postoperatively, B: 2 days postoperatively, C: 1 week postoperatively, D: 8 days postoperatively, E: 10 days postoperatively, F: 2 weeks postoperatively, G: 3 weeks postoperatively, H: 4 weeks postoperatively, I: 6 weeks postoperatively, J: 9 weeks postoperatively, K: 12 weeks postoperatively, L: 6 months postoperatively, M: 12 months postoperatively, N: 24 months postoperatively. (**A**) Network evidence for AOFAS scores; (**B**) Network evidence for VAS scores; (**C**) Network evidence for total postoperative complication rates; (**D**) Network evidence for DVT rates.

The network evidence plots involving 14 intervention times in this NMA, with respect to the AOFAS scores, VAS scores, total postoperative complication rate, and DVT rates, are shown in Figure 3A–D. Lines between two connected points indicate direct comparisons, and unconnected points indicate indirect comparisons *via* NMA. The width of the line represents the number of data sets from the included studies, while the size of the nodes shows the total sample size from A to N.

### AOFAS scores

There were 14 studies reporting AOFAS scores with intervention times including A, B, F, I, J, K, L, M and N. There was a closed ring between the interventions. There were direct and indirect comparisons between the interventions. The consistency test results showed *P* > 0.05. Therefore, the statistical analysis could be performed directly under the consistency model. The results of the NMA showed that the AOFAS scores were lower in B than in L, M and N, lower in F than in K, L, M and N, and lower in I than in L and M. The differences were statistically significant (*P* < 0.05); the remaining comparisons between the two intervention times were not statistically significant (*P* < 0.05) (Table 2).

**Table 2:** Network meta-analysis results of AOFAS scores (RR, 95% CI).

A								
14.56 (−0.78,29.91)	B							
11.61 (−0.70,23.91)	−2.96 (−17.56,11.64)	F						
12.97 (−5.71,31.65)	−1.59 (−19.73,16.55)	1.37 (−15.53,18.26)	I					
−2.24 (−23.20,18.72)	−16.80 (−41.01,7.40)	−13.85 (−36.93,9.24)	−15.21 (−41.68,11.25)	J				
−2.60 (−14.19,8.99)	−17.16 (−31.06,−3.26)	−14.20 (−27.26,−1.15)	−15.57 (−33.13,1.99)	−0.36 (−21.30,20.59)	K			
−7.42 (−18.40,3.56)	−21.99 (−37.10,−6.87)	−19.03 (−32.51,−5.55)	−20.39 (−39.38,−1.40)	−5.18 (−27.52,17.16)	−4.82 (−16.40,6.76)	L		
−10.77 (−23.54,2.00)	−25.33 (−43.25,−7.41)	−22.38 (−38.53,−6.23)	−23.74 (−44.79,−2.69)	−8.53 (−32.18,15.12)	−8.17 (−22.73,6.39)	−3.35 (−16.11,9.41)	M	
−9.44 (−24.51,5.64)	−24.00 (−43.86,−4.15)	−21.05 (−39.23,−2.86)	−22.41 (−45.15,0.32)	−7.20 (−32.34,17.94)	−6.84 (−23.95,10.27)	−2.02 (−17.09,13.05)	1.33 (−14.19,16.85)	N

### VAS scores

There were 10 studies reporting the VAS scores for interventions including A, B, F, G, I, K, L, M and N. The results of the reticulated meta-analysis showed that the VAS scores of B were lower than those of F, G, I, K, L, M and N. The differences were all statistically significant (*P* < 0.05) (Table 3).

**Table 3:** Network meta-analysis results of VAS scores (RR, 95% CI).

A								
27.43 (18.69,36.17)	B							
1.09 (−2.86,5.05)	−26.33 (−35.47,−17.20)	F						
0.39 (−5.00,5.78)	−27.04 (−36.63,−17.44)	−0.70 (−7.02,5.62)	G					
3.25 (−1.52,8.02)	−24.18 (−32.76,−15.60)	2.16 (−2.67,6.98)	2.86 (−3.28,8.99)	I				
−0.81 (−5.02,3.39)	−28.24 (−36.84,−19.64)	−1.91 (−7.52,3.71)	−1.21 (−7.05,4.64)	−4.06 (−10.01,1.88)	K			
1.10 (−2.36,4.56)	−26.33 (−35.42,−17.24)	0.01 (−5.14,5.15)	0.71 (−5.27,6.68)	−2.15 (−7.77,3.46)	1.91 (−2.70,6.53)	L		
1.53 (−1.56,4.62)	−25.90 (−34.75,−17.05)	0.43 (−4.38,5.25)	1.14 (−4.34,6.61)	−1.72 (−6.86,3.41)	2.34 (−1.88,6.56)	0.43 (−3.04,3.89)	M	
1.58 (−2.48,5.63)	−25.85 (−35.25,−16.45)	0.48 (−5.09,6.06)	1.18 (−5.21,7.58)	−1.67 (−7.69,4.34)	2.39 (−2.92,7.70)	0.48 (−3.68,4.63)	0.05 (−4.01,4.11)	**N**

### Total postoperative complication rate

Nineteen studies reported the total postoperative complication rate of the interventions including A, B, C, D, E, F, G, H and I. There was a closed ring between the interventions. There were direct and indirect comparisons between the interventions, and the results of the consistency test showed (*P* > 0.05). Therefore, statistical analysis could be performed directly under the consistency model. The results of NMA showed that there were no statistically significant comparisons between any two interventions (*P* > 0.05) (Table 4).

**Table 4:** Network meta-analysis results of postoperative complication rates (RR, 95% CI).

A								
0.66 (0.08,5.20)	B							
2.36 (0.21,26.20)	3.58 (0.18,72.43)	C						
2.59 (0.23,29.36)	3.92 (0.16,95.00)	1.10 (0.04,33.48)	D					
3.63 (0.21,63.39)	5.50 (0.18,166.11)	1.54 (0.04,57.45)	1.40 (0.03,59.82)	E				
2.08 (0.40,10.95)	3.15 (0.27,36.95)	0.88 (0.06,13.81)	0.80 (0.04,15.25)	0.57 (0.02,13.85)	F			
0.27 (0.05,1.56)	0.40 (0.03,6.12)	0.11 (0.01,2.24)	0.10 (0.01,2.08)	0.07 (0.00,2.12)	0.13 (0.01,1.45)	G		
1.44 (0.15,13.41)	2.18 (0.11,41.92)	0.61 (0.07,5.71)	0.56 (0.02,15.06)	0.40 (0.01,14.05)	0.69 (0.05,10.22)	5.39 (0.31,93.04)	H	
0.86 (0.28,2.60)	1.30 (0.18,9.54)	0.36 (0.04,3.74)	0.33 (0.02,4.78)	0.24 (0.01,4.04)	0.41 (0.08,2.03)	3.21 (0.40,25.89)	0.60 (0.06,6.11)	I

### Incidence of DVT

Eight studies reported the incidence of DVT and interventions including A, B, C, D, E, F, G, H and I. The results of NMA showed that there were no statistically significant comparisons between any two interventions (*P* > 0.05) (Table 5).

**Table 5:** Network meta-analysis results of DVT rates (RR, 95% CI).

A						
0.67 (0.08,5.44)	B					
1.65 (0.05,51.46)	2.47 (0.06,106.26)	C				
1.42 (0.06,35.57)	2.12 (0.06,80.36)	0.86 (0.01,72.70)	E			
0.54 (0.07,4.21)	0.81 (0.05,12.20)	0.33 (0.01,13.90)	0.38 (0.01,14.22)	F		
0.32 (0.03,2.95)	0.48 (0.02,10.13)	0.19 (0.00,11.57)	0.22 (0.00,11.26)	0.59 (0.03,12.11)	G	
0.56 (0.15,2.01)	0.83 (0.11,6.10)	0.34 (0.01,8.17)	0.39 (0.02,8.59)	1.03 (0.14,7.33)	1.75 (0.13,22.76)	H

### Probability ranking of the effect of different postoperative rehabilitation interventions in terms of time

A total of 14 interventions were included in this study. The AOFAS score, VAS score, total postoperative complication rate, and DVT rate were ranked under the 14 interventions. The results of the AOFAS score probability ranking showed that M (85.5%) > N (79.6%) > L (75.4%) >  (58.6%) > J (57.1%) > A (48.2%) > F (17.7%) > I (16.5%) > B (11.5%), suggesting that M may be the intervention time that resulted in the highest AOFAS score in patients. The results of the VAS score probability ranking showed that B (82.8%) > N (75.3%)  > M (66.8%) > L (55.8%) > I (49.3%) > K (36.4%) > G (35.8%) > F (30.6%) > A (17.3%), suggesting that B may be the intervention time that results in the lowest VAS score in patients. The results of the postoperative complication probability ranking showed that E (73.3%) > D (68.8%) > C (67.9%) > F (66%) > H (53.7%) > A (42.4%) > I (35.8%) > B (31.6%) > G (10.5%), suggesting that E may be the intervention time that allows patients to have the lowest total postoperative complication rate. The results of the probability ranking of the incidence of DVT showed that C (66.8%) > E (66%) > A (63.3%) > B (47.6%) > F (40.3%) > H (39%) > G (27.1%), suggesting that C may be the intervention time that resulted in the lowest incidence of DVT in patients.

### Consistency test

The node-splitting method of inconsistency of our results and its Bayesian p value showed good agreement with the confidence intervals crossed with the blank values in all loops (*P* > 0.05), and there were no significant differences between the direct and indirect effects in the network meta-analysis.

### Publication bias

**Figure 4:**
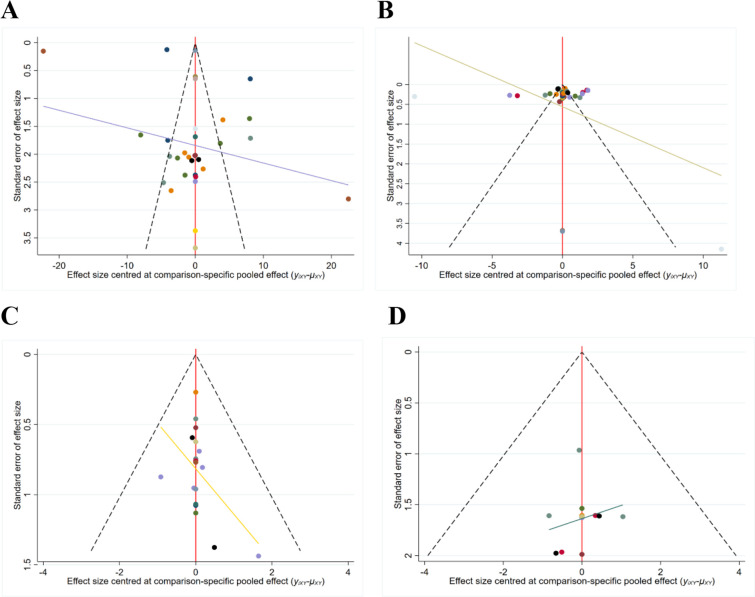
The funnel plot of outcome measures. (**A**) Funnel plot for the AOFAS scores; (**B**) Funnel plot for the VAS scores; (**C**) Funnel plot of the total postoperative complication rates; (**D**) Funnel plot of the DVT rates.

Funnel plots were constructed to analyze publication bias in the included studies (17). However, the scattered distribution of points with incomplete symmetry suggests that there may be some publication bias, and the scattered distribution at the bottom of the funnel plot for each study indicator suggests a small sample effect (Figure 4A–D). Therefore, these results should be interpreted with caution.

## Discussion

The results of this NMA demonstrate our hypothesis that early rehabilitative exercise in the postoperative period was superior to delayed interventions. Rehabilitative exercise, which begins at postoperative day two, is superior for decreasing the VAS score; rehabilitative exercise that begins within 1 week postoperatively may be optimal for reducing the incidence of postoperative complications when it is initiated within 10 days, in order to avoid DVT; and staying in functional rehabilitation exercises for 12 months after surgery may be beneficial for obtaining the best AOFAS score. Overall, based on our confidence interval and ranking results, we conclude that the initiation of rehabilitation on postoperative day 2 may be the best way to improve postoperative pain; that rehabilitation interventions within 10 days after surgery may be optimal for reducing the total incidence of postoperative complications, including DVT; and that continued functional exercise for 12 months after surgery may steadily improve the function of the ankle joint.

Although there is consensus on early rehabilitation with brace protection, the decisions about which postoperative phase (e.g., immediate postoperative period, after wound healing, after scab formation, after fracture healing) to use rehabilitation interventions for patients with peri-ankle fractures vary from surgeon to surgeon. In a 2-part trial performed by Ahl (18, 19), comparing early range of motion (orthosis) and delayed weight bearing, early range of motion and early weight bearing, late range of motion (cast) and delayed weight bearing, or late range of motion and early weight bearing for ankle surgery a trend towards significantly improved functional limb outcomes was found in the early weight bearing, early range of motion group of patients, early motion group that reached statistical significance within 12 months. This study echoes our experimental results perfectly.

For peri-ankle fractures, postoperative functional exercises (including progressive weight bearing, clinical pathway exercises, emerging functional devices, and visual feedback balance exercises) the standard weight-bearing protocol includes 10 to 12 weeks of non–weight bearing followed by 4 to 6 weeks of progressive weight bearing whereby in the first week patients bear weight at 25% of their body weight and increase the amount of weight bearing by 25% each week until they are able to bear their full weight.

A portion of clinicians advocate primary postoperative braking and rehabilitation exercises after wound healing, and they are mainly concerned about the risk of postoperative wound infection. However, this standpoint quickly contradicted that the number of infections was similar in the immediate postoperative and delayed groups (20). Thomas (21) found that early ankle range of motion, with or without early weight bearing, improved range of motion and functional scores at 9 to 12 weeks after treatment. At 1 year, there was no difference between the groups. Despite similar long-term functional outcomes, early motion has been associated with a higher rate of superficial and deep wound complications. The wound complications (e.g., infections and delayed healing) are minimally associated with immediate ankle motion. In most cases, however, patients are fearful of weight-bearing immediately after surgical fixation. Patients' psychological expectations of painful weight-bearing starting within 1 week after surgery are more difficult to manage than in the 4th week after surgery (22), and the bedside physician's education and rehabilitation instructions are particularly important at this time. A biofeedback training study may solve the question of whether 24 h of training complied with touch-down weight-bearing instructions is an effective way to train patients and has wide potential clinical applicability (23).

There is also some concern that weight bearing and activity without waiting for the primary bone callus to form may cause the fracture to displace (24). Laarhoven et al. scholars (25, 26) reported a randomised trial of patients with fractures of the ankle of AO types A, B and C and compared two regimes of postoperative management after internal fixation. They found through long-term follow-up that immediate postoperative weight bearing has not been shown to result in loss of reduction, hardware failure, or revision surgery for osteolysis. Conversely, maintaining some micromovement of the fracture fragment with firm internal fixation may facilitate the healing process (27). Controlled axial loading of the fracture site usually results in a larger volume of healing tissue and a faster healing time compared to no loading or excessive early loading. It has also been suggested that the syndesmotic screws should be removed prior to rehabilitation, because the effect of the syndesmotic screws on ankle mobility may affect the outcome of early rehabilitation training (28). However, there is evidence that removal of the screws does not enhance the ankle range of motion, so whether the screws are removed is also not a criterion for postoperative weight bearing (29). In addition, none of the studies on peri-fractures after fixation allowed unrestricted weight-bearing, generally allowing 20–25 kg of protected weight-bearing. Gul et al. (25) reported on patients with operated Weber A/B/C fractures who were weight-bearing immediately after surgical stabilisation without protective immobilisation treatment for ankle fractures. Their outcomes were better compared to controls who received postoperative plaster immobilisation and non-weight bearing treatment. Patients with early weight-bearing group were found to have significantly less time in hospital, return to work. Under the circumstance of poor fixation or associated with osteoporosis, weight-bearing or exercise needs to be delayed (30). Therefore, the weight-bearing status should be determined by the patient's clinical fracture stability rather than adhering to convention.

Postoperative weight-bearing not only stimulates fracture healing but also nonweight-bearing can be detrimental (31). The consequences of 10 days of bed rest in healthy adults can result in a significant decrease in lower extremity strength, explosive power and aerobic capacity, and reduced physical activity (32). Older adults who undergo postoperative braking are at increased risk for DVT and pulmonary embolism (PE), pneumonia, cardiovascular disease, stressors, and infections (33). According to our findings, these complications (e.g., DVT, PE, etc.) can be minimized by limiting protected functional exercise for 10 days postoperatively.

Although the ultimate goal of surgery is to achieve good joint function, the end of surgery does not mean the end of treatment. Therefore, early postoperative functional exercise should be promoted to all patients with peri-ankle fractures (especially elderly patients) to reduce the restriction on their daily activities, thus shortening the length of hospital stay and returning to independent living as soon as possible (34). Peri-ankle fracture could cause truncal movement asymmetry in the vertical direction accompanied by slower walking cadence and smaller step lengths (35). No studies have been conducted on proprioceptive deficits after peri-ankle fractures and their effect on the rehabilitation process. From our point of view, functional training should be continued for 12 months after surgery to restore proprioception and improve ankle function steadily. Therefore, surgeons and patients should pay attention to the continuity of postoperative rehabilitation exercise.

To our knowledge, this is the first study to explore this aspect using an NMA. However, some limitations that need to be considered may affect the conclusions drawn from this study, such as selection bias due to language limitations, differences in intervention duration, and inadequate reporting of adverse event data. Nevertheless, we believe that the results of this study will help identify the optimal time point for postoperative rehabilitation interventions for peri-ankle fractures and provide further recommendations or guidance for clinical practice.

## Conclusion

We conclude that initiation of rehabilitation on postoperative day 2 may be the best way to improve postoperative pain; that rehabilitation interventions within 10 days after surgery may be optimal for reducing the incidence of postoperative complications, including DVT; and that continued functional exercise for 12 months after surgery may steadily improve ankle joint function. Based on our results, we emphasize the importance of postoperative rehabilitation exercise and suggest the following: first, the appropriate rehabilitation time and protocol should be selected according to the patient's own condition in clinical practice; second, future researchers should make direct comparisons between different rehabilitation times (e.g., within 2 days, 7 days, 7 to 10 days, or other times after surgery); and third, the long-term effects of different start times, such as follow-up surveys at 3, 6, and 12 months after leaving the hospital, should be studied.

## Data Availability

The original contributions presented in the study are included in the article/Suplementary Material, further inquiries can be directed to the corresponding author/s.
